# Microbial Communities Associated with Farmed *Genypterus chilensis*: Detection in Water Prior to Bacterial Outbreaks Using Culturing and High-Throughput Sequencing

**DOI:** 10.3390/ani10061055

**Published:** 2020-06-18

**Authors:** Arturo Levican, Jenny C. Fisher, Sandra L. McLellan, Ruben Avendaño-Herrera

**Affiliations:** 1 Tecnología Médica, Facultad de Ciencias, Pontificia Universidad Católica de Valparaíso, Avenida Universidad 330, Valparaíso 2373223, Chile; 2 Biology Department, Indiana University Northwest, Gary, IN 46408, USA; fisherjc@iun.edu; 3 School of Freshwater Sciences, University of Wisconsin-Milwaukee, Milwaukee, WI 53204, USA; mclellan@uwm.edu; 4 Laboratorio de Patología de Organismos Acuáticos y Biotecnología Acuícola, Facultad de Ciencias de la Vida, Universidad Andrés Bello, Viña del Mar 2571015, Chile; 5 Interdisciplinary Center for Aquaculture Research (INCAR), Concepción 4030000, Chile; 6 Centro de Investigación Marina Quintay (CIMARQ), Universidad Andrés Bello, Casablanca 2480000, Chile

**Keywords:** *Genypterus chilensis*, *Vibrio* spp., *Tenacibaculum* spp., high throughput sequencing, plate counting

## Abstract

**Simple Summary:**

Aquaculture can supplement traditional fisheries to meet the demands of growing populations and may help reduce the overfishing of natural resources. The Chilean Aquaculture Diversification Program has encouraged technological developments for rearing native species such as the red conger eel (*Genypterus chilensis*), but intensive aquaculture practices have led to bacterial outbreaks of *Vibrio* spp. and *Tenacibaculum* spp. in farmed fish. This retrospective study analyzed the natural bacterial community associated with the recirculating seawater used in an experimental *G. chilensis* aquaculture facility to determine if outbreak strains could be identified through regular monitoring. Water samples were analyzed by traditional culturing methods and culture-independent high-throughput amplicon sequencing of the 16S rRNA gene. The sequencing results showed a higher relative abundance of *Vibrio* and *Tenacibaculum* spp. around the time of the outbreaks, but the most abundant genera of cultured isolates did not necessarily match those observed through sequencing. Cultured isolates were assayed for virulence factors and susceptibility to antibiotics to ascertain potential risks and treatments should future outbreaks occur. Although the predominant bacteria identified by the two approaches differed, valuable information was obtained from each method. We thus conclude that the dual monitoring approach is necessary to implement prophylactic measures to prevent future outbreaks.

**Abstract:**

The red conger eel (*Genypterus chilensis*, Guichenot) is a native species included in the Chilean Aquaculture Diversification Program due to high commercial demand. In the context of intensified farming, prior reports link two disease outbreaks with emerging pathogens in the *Vibrio* and *Tenacibaculum* genera. However, the roles remain unclear for the bacterial community and each specific bacterium is associated with the rearing environment for healthy specimens. The success of red conger eel farming therefore warrants research into the bacterial composition of aquaculture conditions and the antimicrobial susceptibilities thereof. This study used culturing methods and high-throughput sequencing to describe the bacterial community associated with water in which *G. chilensis* was farmed. With culturing methods, the predominant genera were *Vibrio* (21.6%), *Pseudolteromonas* (15.7%), *Aliivibrio* (13.7%), and *Shewanella* (7.8%). Only a few bacterial isolates showed amylase, gelatinase, or lipase activity, and almost all showed inhibition zones to commonly-used antibiotics in aquaculture. By contrast, high-throughput sequencing established *Paraperlucidibaca*, *Colwellia*, *Polaribacter*, *Saprospiraceae*, and *Tenacibaculum* as the predominant genera, with *Vibrio* ranking twenty-seventh in abundance. High-throughput sequencing also established a link between previous outbreaks with increased relative abundances of *Vibrio* and *Tenacibaculum*. Therefore, monitoring the presence and abundance of these potential pathogens could be useful in providing prophylactic measures to prevent future outbreaks.

## 1. Introduction

The global aquaculture industry is trending towards diversification, mainly through the farming of native species. The red conger eel (*Genypterus chilensis*, Guichenot) is a bathydemersal fish that inhabits the rocky bottoms of deep-shelf and upper-slope waters in the Southeast Pacific Ocean, with a range extending from southern Peru to southern Chile (https://www.fishbase.de/summary/Genypterus-chilensis.html). The meat of this fish, which is a native Chilean species, is in high gastronomic demand and is only seasonally exploited, primarily by small-scale fishing methods [1]. A sharp decrease in fishing yields over the last 12 years has led to increased prices and an estimated unmet demand of 3048 tons per year. Due to this high demand, new aquaculture programs have concentrated on developing technologies for *G. chilensis* farming [2]. In fact, the red conger eel is currently part of the Chilean Aquaculture Diversification Program (*Programa de Diversificación de la Acuicultura Chilena*).

The intensified aquaculture farming of this fish species has led to the appearance of disease outbreaks across all stages of fish development. For instance, Levican and Avendaño-Herrera [3] identified 21 bacteria associated with massive mortalities of post-larvae *G. chilensis* from a Chilean nursery (33°11′ S 71°1′ W; Quintay Bay, Chile). Most of these bacteria corresponded to *Vibrio* spp., including the classical pathogens *Vibrio anguillarum* and *Vibrio ordalii*, as well as the emerging pathogens *Vibrio tapetis* and *Vibrio toranzoniae* [4]. In fact, *V. tapetis* was also isolated from a specimen that presented vibriosis [5]. In turn, *Tenacibaculum dicentrarchi* infection was reported in a *G. chilensis* brood stock [6]. While mortalities of adult or larval *G. chilensis* specimens in the aforementioned reports were likely due to bacterial infection, unclear roles persist for individual bacterial species isolated from specimens. This lack of clarity is due to poor information on the community composition of bacteria associated with healthy *G. chilensis* specimens [3,6]. Additional studies to determine the composition and antimicrobial susceptibilities of the autochthonous microbiota are needed to improve the success of red conger eel farming [3]. Considering this, a contribution can be made by retrospectively evaluating the bacteria present in the culture water in which the fish and larvae were reared before the reported outbreaks.

One factor that shapes the microbiota composition of reared fish is the environment. When individuals are reared in the same water and with the same diet, the bacterial composition of the tank can mirror the gut microbiota [7]. Understanding the bacterial community composition of the rearing environment is particularly important for larvae production, specifically so that early protective measures can be taken against the threat of fish pathogens [8]. In this regard, comparisons between wild and aquaculture specimens have revealed important differences in microbiota composition. For example, Proteobacteria is the most abundant phylum for wild fine flounder (*Paralichthys adspersus*) and yellowtail kingfish (*Seriola lalandi*), but for aquaculture specimens, the phylum Firmicutes dominates [9,10]. This difference could be explained by variations in the water and host feeding, with a wild microbiota more positively impacting the host than aquaculture-associated microbiota [10].

To date, no studies have examined the microbiota associated with the farming environment of *G. chilensis*, which may contain opportunistic pathogens that could compromise fish health. Knowing which microorganisms tend to associate with these fish in closed aquaculture systems can aid in disease monitoring and prevention, thereby improving the sustainability of the red conger eel farming industry. Considering this, the objective of the present study was to retrospectively determine the composition of the bacterial community associated with the seawater in which *G. chilensis* specimens were farmed, prior to the reported bacterial outbreaks [3,6]. Communities were determined by culturing heterotrophic bacteria and conducting high-throughput sequencing.

## 2. Materials and Methods

### 2.1. Fish Maintenance Conditions

Sampling was carried out between 2014 and 2015 at the Quintay Marine Research Center of the Universidad Andrés Bello (33°13′ S 71°38′ W; Valparaíso Region, Chile). This center maintains an experimental aquaculture system with conditions similar to the wild, as made possible by a recirculating system of fresh, natural seawater. Adult and juvenile red conger eel were kept in 7 m^3^ plastic ponds at a density of 10 ± 2 kg m^−3^. In turn, 20 larvae L^−1^ were kept in 200 L ponds. Adult and juvenile *G. chilensis* were fed with 10 kg of fish or cuttlefish meat once weekly, and no antibiotics were used to prevent/treat infections or stimulate growth. Larvae were fed with live *Artemia nauplii* at a concentration of 1 individual mL^−1^.

Reproductive adults (6.5 ± 0.5 kg weight) and juveniles (1.2 ± 0.1 kg weight) were kept under the following conditions: 35‰ salinity and 12:12 light:dark photoperiod. Larvae were kept under the same conditions, excepting that the photoperiod was 0:24 light:dark, and the water was filtered at 1 µm and with UV light. The overall average seawater temperatures during the summer and winter were 13.5 °C and 11 °C, respectively. However, the average temperature for each sampling month was obtained from records provided by the Chilean Meteorological Service [11,12] for the closest meteorological station (Rodelillo, Viña del Mar, Chile).

This study was carried out in accordance with the principles of the Basel Declaration and recommendations of the Chilean National Commission for Scientific and Technological Research (CONICYT, Chile). Protocols were approved by the Ethics Committee of the Universidad Andrés Bello (Chile) under approval act Nº 021/2013 (dated July 1, 2013).

### 2.2. Seawater Sampling

Analyses were conducted with culture seawater samples collected in 2014–2015, prior to the mortalities described by Levican and Avendaño-Herrera [3]. Culturing and DNA-based methods were used to study the bacteria associated with red conger eel farming. Water samples (500 mL) were collected from each well of the hatchery system in sterile glass bottles. Each well contained fish at different developmental stages. The sampling points have been named as follows: Reproductive Adults, including two types of adults introduced at different times from the natural environment (R, the younger specimens, and RA, the older specimens); first-generation juvenile specimens born in the center (F1); and nursery larvae (N). Samples were collected over the course of a year, specifically the months of March 2014 (from R, RA, F1, N), June 2014 (from RA, F1), October 2014 (from R, F1), December 2014 (from R, F1), and April 2015 (from R, F1). The nursery was only sampled in March 2014 since it closed shortly thereafter because larvae were unavailable due to continuous infectious outbreaks caused by *Vibrio* species [3].

### 2.3. Culturing Methods

To determine cultivable bacteria numbers, serial tenfold dilutions (10^−1^ to 10^−7^) of the seawater samples were prepared in duplicate. Then, 100 µL of the direct samples and each dilution were inoculated onto trypticase soy agar (Oxoid, Basingstoke, UK) supplemented with 1% NaCl (TSA-1), as well as with thiosulfate-citrate-bile-sucrose agar (TCBS, Oxoid, Basingstoke, UK), or Man Rogosa Sharpe agar (MRSA, Difco, Detroit, MI, USA) to obtain a total count of heterotrophic, *Vibrio*, and lactic-acid bacteria, respectively. The inoculated plates were incubated for 48 h to 5 d at 18 °C under aerobic (TSA-1 and TCBS) or microaerophilic conditions (MRSA). Once incubated, the mean number of colonies for each sample was counted on plates with between 30 and 300 colonies. Subsequently, a representative colony was selected of the predominant morphological types in each sample. The selected colonies were re-streaked on TSA-1 agar to obtain pure cultures and were stored in trypticase soy broth (Oxoid, Basingstoke, UK ) supplemented with 1% NaCl and 20% glycerol (Winkler, Lampa, Chile) at −80 °C until further analyses.

To recognize repeated clones in the same sample, all colonies were genotyped by Enterobacterial Repetitive Intergenic Consensus PCR (ERIC-PCR) using the primer pair ERIC-1R and ERIC-2 [13]. PCR products were analyzed on 2% agarose gel, which was stained with 1/10,000 GelRed™ Nucleic Acid Gel Stain (Biotium, San Francisco, CA USA). The GeneRuler™ (ThermoFisher, Waltham, MA, USA) was used as a molecular mass marker, and gels were photographed using the UV transilluminator Gel Doc XR+ (Bio-Rad, Hercules, CA, USA).

A representative isolate from each genotype was identified by amplifying the 16S rRNA gene with the primer pair pA and pH, targeting the nucleotide positions 8-28 and 1542-1522 of the prokaryotic 16S rRNA gene (as represented by *Escherichia coli*) [14]. The expected 1,500 bp amplicons were sequenced using the same primers by Macrogen (Seoul, Korea). The resulting 16S rRNA gene sequences were analyzed with the web-based EzTaxon tool for the identification of prokaryotes [15]. The nucleotide sequences are available in GenBank under accession numbers LS482971-LS483022.

Lytic activities were assessed to determine possible links with the enzymatic capacity of the isolates, which is commonly associated with the ability to cause damage to fish and/or larvae. Lytic activities, i.e., amylase, gelatinase, and lipase activity, were determined as previously described [16]. Moreover, antibiotic susceptibility tests were performed against the most commonly-used drugs in aquaculture, including flumequine, florfenicol, oxolinic acid, oxytetracycline, enrofloxacin, and erythromycin. All analyses were performed following the performance standards for antibiotic susceptibility tests of bacteria isolated from aquatic animals (i.e., VET03 and VET04) [17]. *Aeromonas salmonicida* subsp. *salmonicida* ATCC 33,658 and *E. coli* ATCC 25,922 served as quality control strains.

### 2.4. Culture-Independent Methods

For sequence-based analysis of the relative composition of the community, 200 mL of each water sample were filtered through a 0.45-µm membrane filter (47 mm diameter) (Millipore Corp., Bedford, MA, USA). Filters were immediately frozen and stored at −80 °C.

DNA was extracted from each filter by using the NucleoSpin^®^ Soil Kit (Macherey-Nagel, Dueren, Germany) following the manufacturer’s instructions. The following samples were used: Samples F1, R, and N from March 2014; F1 and RA from June 2014; and samples F1 and R from October 2014, December 2014, and April 2015.

High-throughput amplicon sequencing of the V4-V5 region of the 16S rRNA gene [18] using the MiSeq platform was performed by the Great Lakes Genomics Center (University of Wisconsin-Milwaukee, Milwaukee, WI, USA). Sequence data are available in the NCBI under project SRP156482. Raw, demultiplexed, paired reads were merged using the iu-merge-pairs script from the Illumina Utilities library [19]. The script iu-filter-merged-reads then removed any read pairs with more than three mismatches in the overlapping region. Samples were aligned in mothur [20] using a custom template trimmed to the V4-V5 region, and samples that deviated from the expected size (365–380 nucleotides) were discarded. The classify.seqs function in mothur used the SILVA v. 132 taxonomy database to assign taxonomy to the sequences. Minimum entropy decomposition (MED) analyses [21] were performed for all sequences from the dataset and then separately for sequences associated with two genera of interest, *Tenacibaculum* and *Vibrio*. MED analysis groups samples based on entropy (nucleotide diversity) at specific nucleotide positions to cluster the samples into groups called amplicon sequence variants (ASVs). ASVs are similar to operational taxonomic units, but do not rely on a set threshold for similarity [21]. A minimum abundance criterion (-M) was set for ASVs equal to 0.01% of the total number of sequences for a given analysis (all sequences, *Tenacibaculum*, and *Vibrio*) and maximum variability of nucleotides within a given ASV (-V) of 3 for all analyses. The open-source software for MED analysis, as well as a detailed tutorial for installing and implementing the software, is available at https://github.com/merenlab/oligotyping.

### 2.5. Diversity Analyses

Coverage estimates for all ASVs, ASVs within the genus *Tenacibaculum*, and ASVs within the genus *Vibrio* were calculated using the entropart package [22] in R using the Chao estimator [23]. The coverage estimation provides a measure of how completely the total bacterial community or members of a particular taxon have been identified by sequencing. Shannon entropy was also determined for all samples using diversity in the vegan package [24]. Histograms of ASV abundance showed that a small percentage of ASVs or taxa accounted for the majority of the community. Bar plots for the distribution of the most abundant ASVs by class and genus were generated using base graphics. ASV distributions of the 20 most abundant ASVs within *Tenacibaculum* and *Vibrio* were analyzed by their log-transformed percent abundance within the genus. The pheatmap package [25] was used to display the relative abundance of ASVs within the genus and show hierarchical clustering of samples based on Bray–Curtis dissimilarities.

## 3. Results

### 3.1. Bacterial Abundance

The mean count for cultivable heterotrophic bacteria (TSA-1) was 1.26 × 10^4^ CFU mL^−1^ (median 5.15 × 10^3^ CFU mL^−1^; ranging from 1.60 × 10^3^ CFU mL^−1^ to 8.60 × 10^4^ CFU mL^−1^). The mean concentration of presumptive *Vibrio* spp. (TCBS) was 7.15 × 10^3^ CFU mL^−1^ (median 7.35 × 10^2^ CFU mL^−1^; ranging from 2.00 × 10^0^ CFU mL^−1^ to 8.60 × 10^4^ CFU mL^−1^).

The predominant bacterial species obtained by culturing from TSA-1 and TCBS and identified by 16S rRNA sequencing are shown in Table 1 by date and sampling point. The predominant genera isolated from TSA-1 (total cultivable heterotrophic bacteria) were *Vibrio* (21.6%), *Pseudoalteromonas* (15.7%), *Aliivibrio* (13.7%); *Shewanella* (7.8%), and *Psychromonas* (7.8%). *Vibrio*, *Aliivibrio*, and *Psychrobacter* were the only genera isolated on TCBS. There was no isolation of bacteria from the genus *Tenacibaculum*.

When antibiotic susceptibility tests were carried out for all isolates against flumequine, florfenicol, oxolinic acid, oxytetracycline, enrofloxacin, and erythromycin, most isolates produced inhibition zones around all of the tested antibiotics, and no strains were non-susceptible to multiple classes of antibiotics (Table S1). Only one isolate identified as *Paraperlucidibaca* sp., which was the predominant bacteria in sample F1 from June 2014, did not show inhibition to quinolones flumequine, oxolinic acid, and enrofloxacin.

The enzyme activity of isolates was examined on common substrates as a proxy for potential virulence factors. Only a few isolates showed amylase, gelatinase, or lipase activity, while three (i.e., Q70, Q73, and Q158) showed activity against more than one substrate (Table 1). Different genera showed enzyme activity on the tested substrates, but the most active isolates belonged to the genus *Vibrio*.

### 3.2. High-Throughput Sequence Analysis

**Figure 1:**
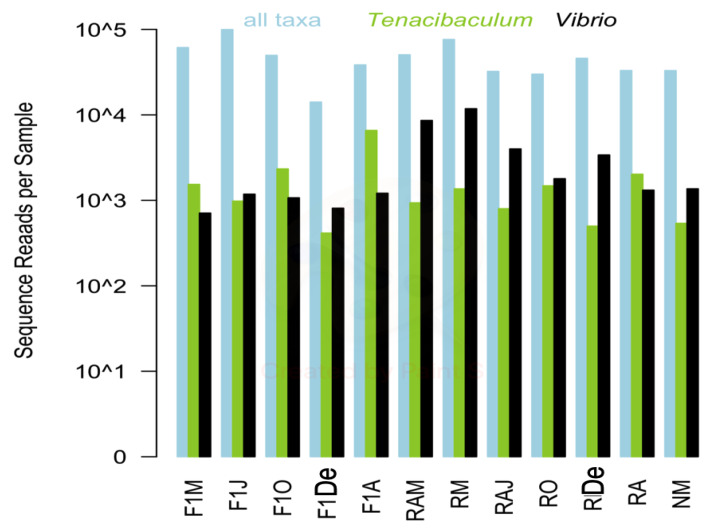
Total bacteria, *Tenacibaculum*, and *Vibrio* sequence reads for each sample are shown in blue, green, and black, respectively. F1M, F1J, F1O, F1De, and F1A: first-generation juvenile specimens born in the center (F1) sampled in March, June, October and December 2014, and April 2015, respectively. RM, RO, RDe, and RA: younger reproductive adults sampled in March, October, and December 2014 and April 2015, respectively. RAM, RAJ: older reproductive adults sampled in March and June 2014, respectively. NM: Nursery larvae sampled in March 2014.

Twelve samples were selected for high-throughput sequencing to provide a full characterization of the microbial community associated with the seawater from a red conger eel farm. A total of 767,745 high-quality sequences were retained for the 12 samples following sequence processing and quality control. Sequences per sample ranged from 21,628 to 114,601; and the abundance of sequences representing the genera *Tenacibaculum* (range: 567 to 7373) and *Vibrio* (710 to 11,819) did not necessarily correlate with overall sequence reads for the sample (Figure 1).

MED analysis grouped samples into ASVs, which are similar to operational taxonomic units, but do not use a fixed similarity (e.g., 97%). MED analysis removed low abundance sequences that may represent either biologically rare organisms or technical “noise” due to sequencing errors. The remaining sequences represented biologically significant organisms consistently found within one or more samples. MED analysis retained 605,907 total sequences, with a range of 16,984 to 99,919 sequences per sample that were grouped into a total of 759 ASVs. Individual ASVs ranged in abundance from 87 to 57,798 sequences (Table S2). MED analyses were run separately to identify key ASVs from the genera *Tenacibaculum* and *Vibrio*. Coverage estimates based on MED ASVs (Table S3) showed that all samples had nearly complete coverage (> 99%) based on all ASVs and ASVs within the genera *Tenacibaculum* (95–99%) and *Vibrio* (91–99%).

Sample diversity based on the Shannon index confirmed that a lower sequencing depth did not limit the detection of key organisms from the sample (Table S4). Shannon diversity based on MED ASVs from all taxa was lowest in the sample with the highest number of sequences reads (i.e., sample F1 from June: 2.72), but diversity was very similar for all other samples (4.83–5.24). Shannon diversity based on the ASVs in *Tenacibaculum* and *Vibrio* ranged from 1.8–4.22 and 1.44–3.04, respectively. Although it seems contradictory that the Shannon diversity could be higher for a single genus than for overall diversity, the index is based on both richness and evenness. Therefore, a sample with a few very dominant MED ASVs would be highly uneven and have a relatively low Shannon diversity.

**Figure 2:**
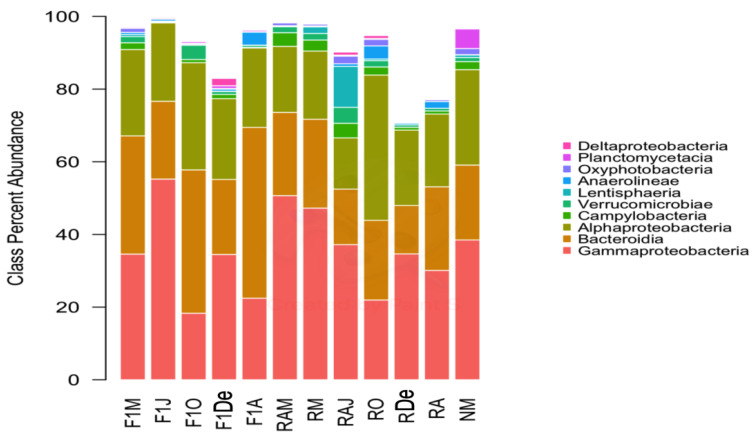
Composition of samples based on the ten most abundant bacterial classes. Colors correspond to the taxa given in the legend. F1M, F1J, F1O, F1De, and F1A: first-generation juvenile specimens born in the center (F1) sampled in March, June, October and December 2014, and April 2015, respectively. RM, RO, RDe, and RA: younger reproductive adults sampled in March, October, and December 2014 and April 2015, respectively. RAM, RAJ: older reproductive adults sampled in March and June 2014, respectively. NM: Nursery larvae sampled in March 2014.

**Figure 3:**
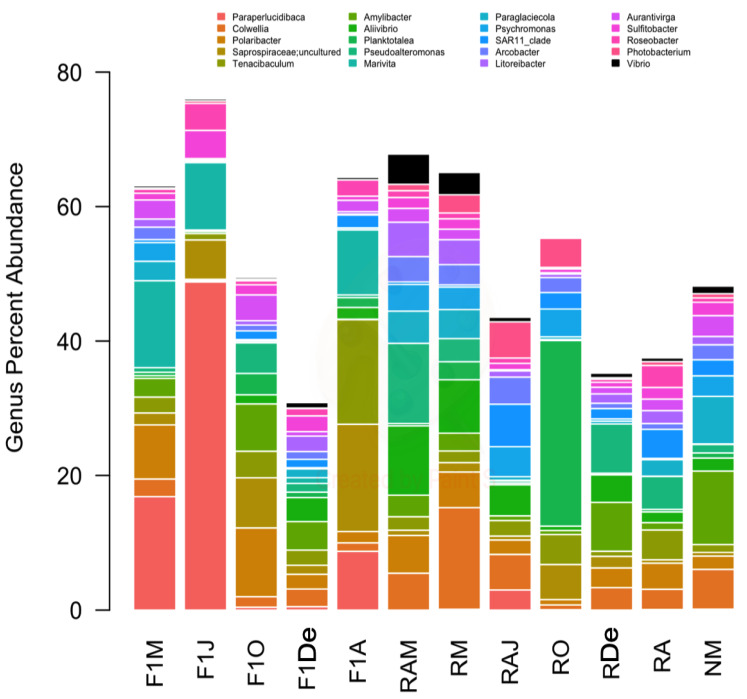
Composition of samples based on the 20 most abundant bacterial genera and the genus *Vibrio*, which was the twenty-seventh most abundant genus. Colors correspond to the genera given in the legend.; *Vibrio* is shown in black. F1M, F1J, F1O, F1De, and F1A: first-generation juvenile specimens born in the center (F1) sampled in March, June, October and December 2014, and April 2015, respectively. RM, RO, RDe, and RA: younger reproductive adults sampled in March, October, and December 2014 and April 2015, respectively. RAM, RAJ: older reproductive adults sampled in March and June 2014, respectively. NM: Nursery larvae sampled in March 2014.

The predominant bacterial classes were Gammaproteobacteria, followed by Bacteroidia, and Alphaproteobacteria (Figure 2). Regarding bacterial genera, *Paraperlucidibaca* showed the highest overall relative abundance, especially in sample F1J, followed by *Colwellia* and *Polaribacter* (Figure 3). The sample that showed the lowest diversity was F1J since the genus *Paraperlucidibaca* represented about 50% of sequences. The *Tenacibaculum* and *Vibrio* genera were the fifth and twenty-seventh most abundant, respectively. Despite having variable relative ASV abundances, the highest values were observed in March 2014 for *Vibrio* spp. and in April 2015 for *Tenacibaculum* spp.

**Figure 4:**
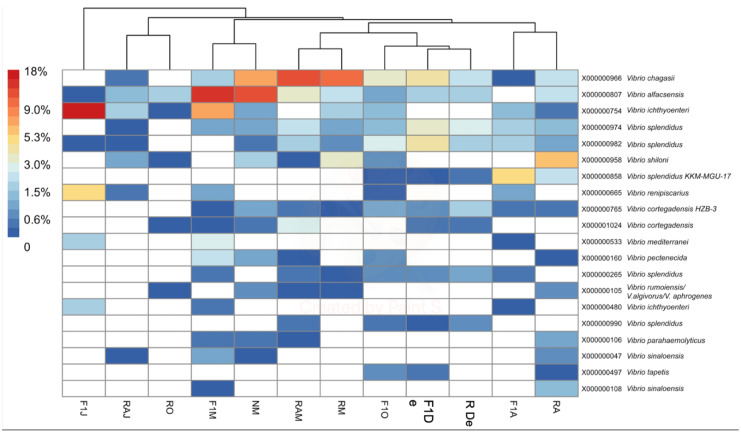
Heatmap of the percent abundance of the 20 most abundant minimum entry decomposition (MED) nodes within the genus *Vibrio*. Hierarchical clustering of samples is based on Bray–Curtis differences among samples calculated using all *Vibrio* nodes (n = 109). F1M, F1J, F1O, F1De, and F1A: first-generation juvenile specimens born in the center (F1) sampled in March, June, October and December 2014, and April 2015, respectively. RM, RO, RDe, and RA: younger reproductive adults sampled in March, October, and December 2014 and April 2015, respectively. RAM, RAJ: older reproductive adults sampled in March and June 2014, respectively. NM: Nursery larvae sampled in March 2014.

**Figure 5:**
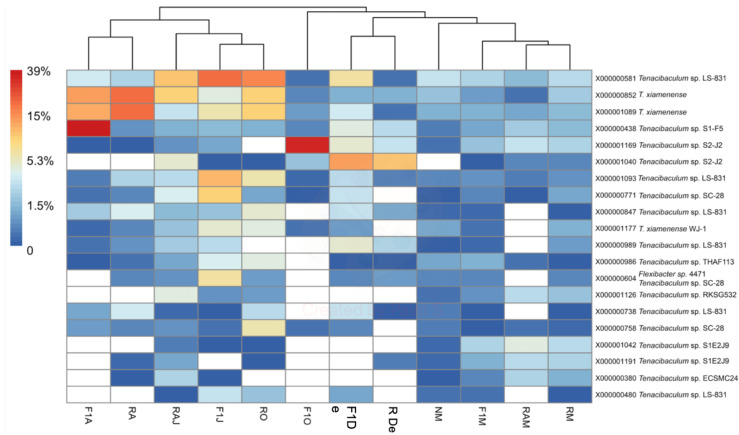
Heatmap of the percent abundance of the 20 most abundant MED nodes within the genus *Tenacibaculum*. Hierarchical clustering of samples is based on Bray–Curtis differences among samples calculated using all *Tenacibaculum* nodes (n = 306). F1M, F1J, F1O, F1De, and F1A: first-generation juvenile specimens born in the center (F1) sampled in March, June, October and December 2014, and April 2015, respectively. RM, RO, RDe, and RA: younger reproductive adults sampled in March, October, and December 2014 and April 2015, respectively. RAM, RAJ: older reproductive adults sampled in March and June 2014, respectively. NM: Nursery larvae sampled in March 2014.

To determine the identity of organisms represented by ASVs within both genera, BLASTn analysis (National Center for Biotechnology Information, https://blast.ncbi.nlm.nih.gov) was performed for the ASV representing each of the 20 most abundant MED nodes (Figure 4 and Figure 5). The criterion used for identification was a > 98% match with available sequences from isolates of known species. In the case of *Vibrio* ASVs, the most abundant species represented were *Vibrio chagasii*, *Vibrio alfacsensis*, *Vibrio ichthyoenteri*, and *Vibrio splendidus* (Figure 4). In the case of *Tenacibaculum*, ASVs matched mostly uncultured bacteria, with the only recognized species being *Tenacibaculum xiamenense* (Figure 5). A local BLASTn search conducted with ASVs, using 16S rRNA gene sequences of the isolate as a database, showed that most isolates with full-length sequences (40/49) shared > 97% sequence similarity with an ASV. Two of the isolates had only partial 16S rRNA that did not span the V4V5 region and, thus, could not be matched to an ASV. 

## 4. Discussion

This retrospective study is the first to examine microbial communities associated with the seawater of red conger eel aquaculture. Therefore, no direct comparisons for operations farming this particular fish species were available. However, the abundance of total heterotrophic bacteria was similar to that previously reported for natural seawater used for aquaculture systems rearing puffer fish (*Takifugu rubripes*) and ayu (*Plecoglossus altivelis*) [8]. In this study, cultivable *Vibrio* spp. represented about 25% of the isolated bacteria, while previous estimates were only 1.7–2.6% [8]. These differences could be due to the species being farmed, the season, or the feeding system.

Although Gammaproteobacteria were the predominant bacterial class obtained by culturing and high-throughput sequencing, the predominant genus detected by each approach was different. For TSA-1 and TCBS cultures, *Vibrio* was the predominant genus, but *Vibrio* ranked twenty-seventh in abundance using high-throughput sequencing. By contrast, no *Tenacibaculum* species were isolated, but this genus was detected by genomic analysis. These discrepancies are common in environmental samples due to (i) the low proportion of bacteria that can be grown in pure cultures under laboratory conditions and (ii) competition between different bacterial groups for the culture medium. Unfortunately, *Tenacibaculum* members grow only in media prepared with seawater (e.g., *Flexibacter maritimum* or marine agar) [6,26]. Such media were not used in this study because, at the time of sampling, *Tenacibaculum* was not yet reported as a pathogen of conger eel, nor was *Tenacibaculum* reported in this center. Furthermore, since TCBS is selective for *Vibrio* culturing, it is not surprising that a high number of *Vibrio* spp. and closely related genera grew on this medium. In marine environments, cultivable organisms represent about 1% of the total community [9,10].

Culture-independent approaches, such as high-throughput sequencing, provide a more complete view of microbiota composition by providing deep coverage that far exceeds the limits of bacterial recovery from the culture medium [9,10]. In fact, cultivable heterotrophic bacteria in this study did not represent the whole diversity detected by high-throughput sequencing; however, it has been stated that microbe cultivation is essential for understanding the close physiological relationship between the host and microbiota isolates [27]. Despite culturing results being less representative of bacterial-community composition than those for high-throughput sequencing, the parallel use of both approaches is still recommended. Doing so in the present study provided a proper characterization of the physiological characteristics, antibiotic susceptibility, and other relevant traits of the assessed bacteria. In the current case, most isolates (82%) shared a high level of identity with a representative ASV, suggesting that most of the obtained isolates are also relatively abundant in the environment.

The diversity of bacterial communities in the present study was similar to that observed in wild *P. adspersus* and *S. lalandi* by Ramirez and Romero [9,10], with the predominant bacterial genera belonging to the Gammaproteobacteria class. Ramirez and Romero [9,10] indicated that the establishment of different microbiota between wild and aquaculture specimens was related to various environmental parameters, such as water quality, population density, and habitat. In the present study, farmed red conger eels were fed fish or cuttlefish meat, which is similar to a wild diet. Differences in microbiota could be strongly related to host feeding, and microbiota associated with a wild origin may provide more positive characteristics for the host than aquaculture-associated bacteria [10]. In this same line, the predominant genera detected in the current study (i.e., *Pseudoalteromonas*, *Aliivibrio*, *Shewanella*, *Psychromonas*, and *Photobacterium*), with the exception of *Vibrio* spp., have previously been associated with beneficial characteristics, such as enhancing immune parameters and improving survival against vibriosis in *Solea senegalensis* [10].

Overall, predominant bacteria isolated by culturing in this study showed low lytic capacity in different substrates (Table 1), suggesting a lack of key virulence factors common to pathogenic strains, such as hydrolytic enzymes, which are implicated in the pathogenesis of marine microorganisms [28]. Furthermore, almost all isolates showed inhibition zones against commonly-used antibiotics in aquaculture systems, whereas only one isolate did not show inhibition zones to multiple quinolones (i.e., *Paraperlucidibaca* sp.). These findings suggest that any disease outbreaks occurring due to opportunistic bacteria common to the microbiota could be treated with antibiotic therapy. This high susceptibility was expected since the assessed aquaculture system had a continuous input of fresh seawater and no history of antibiotics use.

Considering that *Vibrio* spp. have been associated with mass mortalities of post-larvae red conger eel at the Quintay Marine Research Center [3] and, more recently, that *T. dicentrarchi* has been reported in an epizootic outbreak among brood stock of red conger eel [6], a deep analysis via high-throughput sequencing was performed on both genera to provide a more complete view of the bacterial composition present in seawater. While ASVs could be too short to obtain a reliable identification at the species level by BLASTN analysis, the sequences representing the 20 most abundant MED nodes from *Vibrio* and one from *Tenacibaculum* were identified. These sequences showed matches between 98% and 100% with available sequences of a unique known species (e.g., *T. xiamenense*). It must be acknowledged, however, that the sequences could belong to other species with high 16S rRNA identities to the determined species.

*Vibrio* and *Tenacibaculum* were present in all samples studied, with no clear seasonality. However, variations at the species level could be associated with temperature changes (Table 1). At the genus level, high-throughput sequencing revealed two instances of increased relative abundance (Figure 1). Interestingly, both cases of increased relative abundance can be linked to an outbreak of the respective genera. For *Vibrio* spp., the relative increase was observed in March 2014, and the predominant species detected were *V. chagasii*, *V. alfacsensis*, *V. ichthyoenteri*, and *V. splendidus* (F1, N, RA, and R from March 2014; Figure 4). This relative increase was associated with an outbreak of vibriosis among some adult red conger eel in May 2014. Water samples were not obtainable exactly during the outbreak since healthy specimens were being treated with a prophylactic treatment of formalin. However, two weeks later, in June 2014 (samples F1 and RA), a higher relative abundance of *V. ichthyoenteri* was found. Additionally, found with higher relative abundances, but lower than that of *V. ichthyoenteri*, were *V. chagasii*, *V. alfacsensis*, and *V. splendidus* (Figure 4). Another pathogenic species for maricultured animals, *V. alginolyticus* (considered one of the most abundant *Vibrio* spp. in seawater [29]), was neither isolated by plate culture nor included among the most abundant MED nodes within the genus *Vibrio*. This could be attributed to the fact that *V. alginolyticus* has been previously found in temperate water facilities, and water temperatures at the at the Quintay Marine Research Center are cold (about 20 °C; Table 1).

While water samples were not collected during the outbreak, a moribund specimen was sampled, and *V. tapetis* was isolated from external gross lesions in the mouth and from the aqueous humor of the eyes [5]. Additionally, *V. ichthyoenteri* was isolated from the kidney of the moribund specimen (unpublished results). The obtained *V. tapetis* isolates, together with others recovered from diseased fine flounders reared at the Quintay Marine Research Center, were characterized by Levican et al. [5], and the new subspecies *V. tapetis* subsp. *quintayensis* was described. A representative isolate was injected intraperitoneally into a fine flounder specimen, but neither disease nor mortality resulted. However, opportunistic pathogenicity could not be ruled out [5]. Moreover, *V. tapetis* in combination with *V. ichthyoenteri* might possibly have produced vibriosis in the diseased specimens. This hypothesis would be supported by descriptions of *V. ichthyoenteri* being first isolated from the kidney of a moribund Atlantic salmon (*Salmo salar*) smolt held in seawater [30], in addition to associations with vibriosis, particularly in small olive flounder (*Paralichthys olivaceus*) [31].

Although cultured species of *Tenacibaculum* were not identified by high-throughput sequencing, an increase in relative abundance was observed in April 2015 (Figure 5). In July 2015, when the above-mentioned outbreak occurred, *T. dicentrachi* was isolated from gross lesions and inner organs [6]. The authors concluded that while the recorded red conger eel mortalities might be due to bacterial infection, the commensal or opportunistic role of *T. dicentrarchi* remains unclear. Aligning with the present study, high-throughput sequencing recently detected *Tenacibaculum* spp. in Atlantic salmon as associated with parasitism by sea lice (*Lepeophtheirus salmonis*) [32], as well as in the microbiome of Antarctic killer whales (*Orcinus orca*) [33]. Despite not being able to link *Tenacibaculum* spp. with pathogenicity, Llewellyn et al. [32] concluded that perturbation of the mucosal microbiome might promote pathology via decreased colonization resistance to exogenous opportunists and/or the proliferation of endogenous pathogenic genera, including *Tenacibaculum*. In turn, Hooper et al. [33] reported a significant correlation between diatom and *T. dicentrarchi* abundances, where samples with high *T. dicentrarchi* abundances showed distinct functional profiles that could be linked with individual risk; nevertheless, more research is needed to verify this possible link.

The Quintay Marine Research Center is a small experimental center with only a few wells dedicated to raising red conger eels. These wells were sampled repeatedly during the 2014–2015 year, but the limited number of samples collected may not completely represent the bacterial diversity associated with the rearing environment. However, this is the first study to assess the bacterial community associated with an aquaculture facility for this fish species, and the combination of culturing and culture-independent approaches provide preliminary results that could be useful in the design of future studies.

## 5. Conclusions

The obtained results link the composition of microbial communities found in the red conger eel environment to two outbreaks caused by an increase in the relative abundances of *Vibrio* and *Tenacibaculum* species, respectively. High-throughput sequencing tools demonstrated to be useful in identifying potentially infectious bacteria within farming systems, whereas bacterial isolation can be of help in laying the foundation for possible outbreak treatments. Therefore, the use of both approaches in monitoring the presence and abundance of these potential pathogens will be useful in providing prophylactic measures to prevent future outbreaks.

## Figures and Tables

**Table 1:** Characteristics of isolates recovered in this study.

Sample	Temp Max	ID Number	Genus	Species	Isolation Media	LIP	GEL	AMY
**F1 Mar**	21.7 °C March 2014	Q24	*Pseudoalteromonas*	*P. carragenovora*	TSA-1	-	+	-
		Q25	*Pseudoalteromonas*	*P. undina*	TSA-1	-	+	-
		Q27	*Psychrobacter*	*P. piscatori*	TCBS	-	-	-
**RA Mar**	21.7 °C March 2014	Q1	*Pseudoalteromonas*	*P. espejiana*	TSA-1	-	+	-
		Q2	*Pseudoalteromonas*	*P. espejiana*	TSA-1	-	-	-
		Q3	*Aliivibrio*	*A. logei*	TCBS	-	-	-
**R Mar**	21.7 °C March 2014	Q12	*Psychromonas*	*P. ingahanii*	TSA-1	-	-	-
		Q14	*Shewanella*	*S. pacifica*	TSA-1	-	+	-
		Q15	*Photobacterium*	*P. aquimaris*	TSA-1	-	-	-
		Q16	*Vibrio*	*V. cortegadensis*	TCBS	-	-	-
**N Mar**	21.7 °C March 2014	Q38	*Vibrio*	*V. hemicentroti*	TSA-1	-	+	-
		Q39	*Idiomarina*	*I. loihensis*	TSA-1	-	+	-
		Q40	*Alteromonas*	*Alteromonas sp.*	TSA-1	-	-	-
**F1 June**	14.8 °C June 2014	Q64	*Vibrio*	*V. lentus*	TSA-1	+	-	-
		Q65	*Alivibrio*	*A. sifiae*	TSA-1	+	-	-
		Q66	*Shewanella*	*S. pneumatophori*	TSA-1	+	-	-
		Q67	*Alivibrio*	*A. sifiae*	TCBS	-	-	-
		Q68	*Paraperlucidibaca*	*P. baekdonensis*	TSA-1	-	-	-
		Q69	*Alteromonas*	*A. australica*	TSA-1	+	-	-
**RA June**	14.8 °C June 2014	Q59	*Pseudoalteromonas*	*P. arctica*	TSA-1	+	-	-
		Q60	*Shewanella*	*S. sairae*	TSA-1	+	-	-
		Q61	*Psychromonas*	*P. arctica*	TSA-1	-	-	-
		Q62	*Alivibrio*	*A. sifiae*	TCBS	+	-	-
**F1 Oct**	18.9 °C October 2014	Q77	*Vibrio*	*V. gallaecicus*	TSA-1	-	-	-
**RA Oct**	18.9 °C# October 2014	Q70	*Vibrio*	*V. hemicentroti*	TSA-1	-	+	+
		Q71	*Pseudoalteromonas*	*P. arctica*	TSA-1	+	-	-
		Q72	*Shewanella*	*S. schegeliana*	TSA-1	-	+	-
		Q73	*Bacillus*	*B. hwajinpoensis*	TSA-1	-	+	+
		Q74	*Bacillus*	*B. thioparans*	TSA-1	-	-	+
		Q75	*Paraperlucidibaca*	*P. baekdonensis*	TSA-1	-	-	-
**R Oct**	18.9 °C October 2014	Q76	*Bacillus*	*B. toyonensis*	TSA-1	-	+	-
**F1 Dec**	21.2 °C December 2014	Q101	*Vibrio*	*Vibrio sp.*	TSA-1	-	-	+
		Q102	*Vibrio*	*V. lentus*	TSA-1	-	-	+
		Q103	*Vibrio*	*V. splendidus*	TSA-1	-	-	+
		Q104	*Vibrio*	*Vibrio sp.*	TSA-1/TCBS	-	-	+
**R Dec**	21.2 °C December 2014	Q94	*Aliivibrio*	*A. finisterrensis*	TSA-1	-	-	-
		Q95	*Photobacterium*	*P. piscicola*	TSA-1	+	-	-
		Q96	*Alivibrio*	*A. sifiae*	TSA-1	+	-	-
		Q97	*Vibrio*	*V. tapetis*	TSA-1	-	-	-
		Q98	*Vibrio*	*V. gigantis*	TCBS	-	-	-
**F1 Apr**	21.3 °C April 2015	Q156	*Psychromonas*	*P. aquatilis*	TSA-1	-	-	-
		Q157	*Psychrobacter*	*P. proteolyticus*	TSA-1	-	-	-
		Q158	*Photobacterium*	*P. sanguinicancri*	TSA-1	+	-	+
		Q165	*Pseudoalteromonas*	*P. marina*	TSA-1	-	-	-
		Q168	*Vibrio*	*V. splendidus*	TCBS	-	-	+
		Q160	*Acinetobacter*	*A. ursingii*	TSA-1	-	-	-
**R Apr**	21.3 °C April 2015	Q151	*Alivibrio*	*A. logei*	TSA-1	-	-	-
		Q152	*Photobacterium*	*P. aquimaris*	TSA-1	-	-	-
		Q153	*Acinetobacter*	*A. lwofii*	TSA-1	-	-	-
		Q155	*Vibrio*	*V. splendidus*	TCBS	-	-	-

Temp Max, Average of Max. temperature during sampling month; LIP: Lipase activity; GEL: Gelatinase activity; AMY: Amylase activity. TSA-1: trypticase soy agar supplemented with 1% NaCl. TCBS: thiosulfate-citrate-bile-sucrose agar. F1: first-generation juvenile specimens born in the center. R: younger reproductive adults. RA: older reproductive adults. N: Nursery larvae.

## Supplementary Materials

The following are available online at https://www.mdpi.com/2076-2615/10/6/1055/s1, Table S1. Antibiotic susceptibility of the isolates. Table S2. Number of sequences per sample retained or removed by MED analysis. Table S3. Coverage estimates for MED nodes within all taxa, *Tenacibaculum*, and *Vibrio*. Table S4. Shannon Diversity index for MED nodes within all taxa, *Tenacibaculum*, and *Vibrio*.
