# Supplementary material for: Microbial Communities Associated with Farmed Genypterus chilensis: Detection in Water Prior to Bacterial Outbreaks Using Culturing and High-Throughput Sequencing
Source: Animals (Basel). 2020 Jun 18;10(6):1055. doi: 10.3390/ani10061055 (PMC7341507; doi:10.3390/ani10061055)
Supplement: Supplementary file 1 [file animals-10-01055-s001.pdf]

Supplementary Table 1. Antibiotic susceptibility of the isolates

UB, Flumequine (>30mm: Wild type; <30mm: Non-Wild type); FFC, Florfenicol (>27mm: Wild type; <26mm: Non-Wild type); OA, Oxolinic acid (>30: Susceptible; 25-25: Intermediate; <24: Resistant); OX-T, Oxytetracycline (>28: Susceptible; 22-27: Intermediate; <21: Resistant); ENR, Enrofloxacin (>30: Susceptible; 25-25: Intermediate; <24: Resistant); ERI, Erythromycin (>14mm: Wild type; <13mm: Non-Wild type).

Sample		UB	FFC	OA	OX-T	ENR	ERI
F1 Mar	Q24	52	41	47	44	54	49
	Q25	48	40	50	47	57	46
	Q27	43	38	45	33	48	30
F1 Jun	Q63	34	41	30	41	35	21
	Q64	35	39	30	39	34	24
	Q65	33	35	29	34	31	21
	Q66	40	34	35	27	37	26
	Q67	37	34	32	35	32	14
	Q68	28	29	6	56	6	33
	Q69	38	33	33	26	28	26
F1 Oct	Q77	49	48	42	48	43	31
F1 Dec	Q101	60	60	60	60	60	60
	Q102	32	38	29	36	31	16
	Q103	35	38	30	37	33	18
	Q104	60	63	40	48	66	38
F1 Apr	Q156	60	47	67	54	60	42
	Q157	52	47	51	40	50	39
	Q158	38	36	30	35	33	18
	Q165	45	35	37	29	39	30
	Q168	60	60	60	60	60	60
	Q160	38	36	28	28	41	6
RA Mar	Q1	46	48	60	46	55	30
	Q2	58	46	50	41	56	45
	Q3	35	44	38	45	40	25
R Mar	Q12	36	54	41	40	53	6
	Q14	51	41	38	37	46	26
	Q15	30	25	27	26	30	16
	Q16	33	38	24	39	30	22
RA Jun	Q59	56	41	47	34	51	42
	Q60	42	31	33	26	38	27
	Q61	53	50	46	49	59	73
	Q62	35	33	33	29	29	20
RA Oct	Q70	33	37	29	35	35	19
	Q71	44	35	37	29	39	30
	Q72	42	36	32	29	37	28
	Q73	39	35	40	37	35	38
	Q74	28	36	35	36	38	36
	Q75	60	60	60	60	60	60
R Oct	Q76	31	34	30	27	31	28
R Dec	Q94	58	44	54	43	56	32
	Q95	41	39	36	35	37	19
	Q96	55	50	48	43	44	28
	Q97	60	60	60	60	60	60
	Q98	55	43	59	60	48	20
R Apr	Q151	41	34	36	32	35	18
	Q152	35	34	33	35	31	12
	Q153	44	35	34	32	39	15
	Q155	33	33	27	31	27	14
N Mar	Q38	33	31	28	30	27	15
	Q39	65	43	47	40	62	26
	Q40	49	40	45	43	70	59

Supplementary Table 2. Number of sequences per sample retained or removed by MED analysis.

Sample	Retained	Removed by -M[a]	Removed by -V[b]
F1J	99919	10644	4038
F1A	39920	6460	2027
F1O	52774	9477	2849
F1M	62474	15589	5838
F1Di	16984	3485	1159
RAM	51062	10973	3935
RAJ	33895	11374	3463
RA	42486	7378	2641
RM	77082	17740	6354
RO	30973	7410	2465
RDi	64552	7605	2666
NM	33786	12490	3777

[a] Sequences in nodes removed because total abundance of node was below minimum threshold
[b]Sequences removed because nucleotide variability was too high to allow placement within a node

Supplementary Table 3. Coverage estimates for MED nodes within all taxa, *Tenacibaculum*, and *Vibrio*.

	**Coverage**[a]		
**Sample**	**all taxa**	***Tenacibaculum***	***Vibrio***
F1M	>0.999	0.975	0.959
F1J	0.999	0.966	0.995
F1O	0.999	0.988	0.908
F1Di	0.996	0.947	0.909
F1A	0.999	0.997	0.944
RAM	0.999	0.950	0.997
RM	>0.999	0.969	0.997
RAJ	0.999	0.953	0.942
RO	0.998	0.975	0.913
RDi	0.999	0.954	0.978
RA	0.999	0.994	0.971
NM	0.999	0.950	0.969

[a]Coverage estimates were calculated with the *entropart* package in R using the Chao estimator.

Supplementary Table 4. Shannon Diversity index for MED nodes within all taxa, *Tenacibaculum*, and *Vibrio*

	Shannon Diversity[a]		
**Sample**	**all taxa**	***Tenacibaculum***	***Vibrio***
F1M	4.85	4.22	2.37
F1J	2.72	3.21	1.44
F1O	4.83	1.80	3.04
F1Di	4.94	3.29	2.71
F1A	4.22	2.23	2.44
RAM	5.09	4.00	2.56
RM	5.07	4.20	2.58
RAJ	5.1	3.96	2.55
RO	4.55	3.40	2.75
RDi	4.58	3.23	3.04
RA	5.24	2.66	2.95
NM	4.99	3.94	2.38

[a]Shannon diversity was calculated using the *vegan* package in R.
